# Distinct protein patterns related to postnatal development in small for gestational age preterm infants

**DOI:** 10.1038/s41390-024-03481-0

**Published:** 2024-08-16

**Authors:** Eva R. Smit, Michelle Romijn, Pieter Langerhorst, Carmen van der Zwaan, Hilde van der Staaij, Joost Rotteveel, Anton H. van Kaam, Suzanne F. Fustolo-Gunnink, Arie J. Hoogendijk, Wes Onland, Martijn J. J. Finken, Maartje van den Biggelaar

**Affiliations:** 1 Department of Molecular Hematology, Sanquin Research, Amsterdam, the Netherlands; 2 Department of Neonatology, Amsterdam UMC location University of Amsterdam, Amsterdam, the Netherlands; 3 Amsterdam Reproduction and Development Research Institute, Amsterdam, the Netherlands; 4 Department of Pediatric Endocrinology, Amsterdam UMC location Vrije Universiteit Amsterdam, Amsterdam, the Netherlands; 5 Sanquin Research & Lab Services, Sanquin Blood Supply Foundation, Amsterdam, the Netherlands; 6 Department of Pediatrics, Division of Neonatology, Willem-Alexander Children’s Hospital, Leiden University Medical Center, Leiden, the Netherlands; 7 Department of Pediatric Hematology, Emma Children’s Hospital, Amsterdam University Medical Center, Amsterdam, the Netherlands

## Abstract

**Background:**

Preterm infants, especially those born small for gestational age (SGA), are at risk of short-term and long-term health complications. Characterization of changes in circulating proteins postnatally in preterm infants may provide valuable fundamental insights into this population. Here, we investigated postnatal developmental patterns in preterm infants and explored protein signatures that deviate between SGA infants and appropriate for gestational age (AGA) infants using a mass spectrometry (MS)-based proteomics workflow.

**Methods:**

Longitudinal serum samples obtained at postnatal days 0, 3, 7, 14, and 28 from 67 preterm infants were analyzed using unbiased MS-based proteomics.

**Results:**

314 out of 833 quantified serum proteins change postnatally, including previously described age-related changes in immunoglobulins, hemoglobin subunits, and new developmental patterns, e.g. apolipoproteins (APOA4) and terminal complement cascade (C9) proteins. Limited differences between SGA and AGA infants were found at birth while longitudinal monitoring revealed 69 deviating proteins, including insulin-sensitizing hormone adiponectin, platelet proteins, and 24 proteins with an annotated function in the immune response.

**Conclusions:**

This study shows the potential of MS-based serum profiling in defining circulating protein trajectories in the preterm infant population and its ability to identify longitudinal alterations in protein levels associated with SGA.

**Impact:**

- Postnatal changes of circulating proteins in preterm infants have not fully been elucidated but may contribute to development of health complications.
- Mass spectrometry-based analysis is an attractive approach to study circulating proteins in preterm infants with limited material.
- Longitudinal plasma profiling reveals postnatal developmental-related patterns in preterm infants (314/833 proteins) including previously described changes, but also previously unreported proteins.
- Longitudinal monitoring revealed an immune response signature between SGA and AGA infants.
- This study highlights the importance of taking postnatal changes into account for translational studies in preterm infants.

## Introduction

Preterm birth (<37 weeks GA) is a leading cause of mortality in infants and children under five years, with higher risks for those infants born more prematurely.^1^ In 2019, this resulted in approximately 900,000 deaths worldwide.^2,3^ Preterm birth is also associated with a high risk of short-term effects on health, including necrotizing enterocolitis, sepsis, bronchopulmonary dysplasia (BPD), and retinopathy of prematurity. In addition, preterm infants face various health risks at a later age, such as neurodevelopmental impairment, poorer growth, and type 2 diabetes mellitus.^4–7^ Those infants born small for gestational age (SGA) are generally at higher risks for these diagnoses.^7–11^

Timely clinical interventions are critical to minimize potential long-term morbidities in infants that develop as a result of complications during their NICU stay should they occur.^12^ To this end, preterm infants are continuously monitored using purposive sampling for measurement of blood-based markers such as C-reactive protein (CRP), bacterial cultures, and cell counts. However, the whole blood volume required for these laboratory tests is considerable compared to the total blood volume in neonates (~70 mL/kg).^13^ Importantly, substantial iatrogenic blood loss is associated with an increased risk of anemia.^14^ As a result, additional blood draws for basic research to improve fundamental understanding of common health complications in preterm infants are limited.

Whole blood contains hundreds of proteins^15^ that are currently not measured in standard monitoring. These proteins can provide insight into a wide range of major biological processes, including metabolic state, inflammation, complement system, hemostasis, and both the adaptive and immune system^15^ that is currently not attained in standard diagnostic clinical care. These proteins may contribute to a better basic understanding of preterm infants. Previous studies focused on either the impact of gestational age (GA) on protein levels in cord blood or postnatal protein changes in full-term infants and have found major changes in hemoglobin composition, immunoglobulins, energy homeostasis systems, and hormonal axes after birth.^16–25^ However, a recent study showed that most protein changes in the first month after birth of extremely preterm infants are associated with postnatal age instead of GA.^26^ This suggests that translational studies investigating the development of morbidities in this population require an understanding of how these circulating proteins change postnatally in order to identify deviations associated with their morbidities of interest.

Due to its unbiased nature and small plasma volume requirement per assay mass spectrometry (MS) based profiling is an attractive tool to study this vulnerable population of preterm infants.^15^ Recent advances in the field of MS-based serum profiling have led to high-throughput workflows, thereby providing a plethora of information on a wide range of biological processes from one sample. Here, we explored the potential of MS-based serum proteomics in the systematic and unbiased evaluation to investigate age-related changes in circulating protein levels and biological processes in preterm infants.

## Methods

### Study samples from preterm infants

**Fig. 1:**
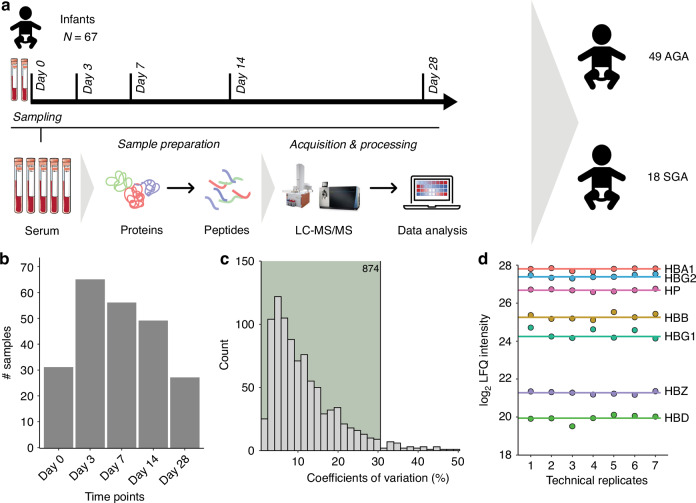
PRIDICT-BPD study design and serum proteomics. **a** Overview of the PRIDICT-BPD cohort and blood sample collection, consisting of 49 appropriate for gestational age (AGA) and 18 small for gestational age (SGA) infants. Samples collected from infants and healthy adults were analysed using the proteomics workflow starting with serum sample preparation, followed by LC-MSMS analysis and data analysis. **b** Overview of the samples collected in cord blood (day 0) and from capillary or arterial blood (day 3, day 7, day 14, and day 28) in the PRIDICT-BPD cohort. **c** Histogram representing the coefficient of variation (CV) for all proteins quantified across repeated injections of a study wide quality control sample, with 874 proteins reliably quantified with high reproducibility (CV < 30%). **d** Label free quantification (LFQ)-intensity levels plotted for quantified haemoglobin subunits (zeta: HBZ; alpha: HBA1; beta: HBB; delta: HBD; gamma: HBG1/HBG2) and haptoglobin (HP) proteins across the technical replicates.

Samples from preterm infants were obtained from the PulmonaRy Inflammation and glucocorticoiD sensitivity for the PReDICTion of BronchoPulmonary Dysplasia (PRIDICT-BPD) study, addressing the feasibility of various biomarkers for the prediction of BPD in a unselected population of preterm infants.^27^ For this specific study, left-over serum samples were used from participants if caregiver(s) gave written informed consent for usage of study material for future research. The PRIDICT-BPD study was performed between November 2019 and October 2021 at the two NICUs of the Amsterdam University Medical Center in the Netherlands. Infants were eligible to participate in this study if they were born at a GA below 30 weeks, and had no major congenital malformations. The Medical Research Ethics Committee of Vrije Universiteit Amsterdam approved the study (protocol number 2019.371). In the PRIDICT-BPD study, we collected cord blood samples at birth and capillary or arterial blood samples at postnatal days 3, 7, 14, and 28, as long as the participants were admitted to the NICU (Fig. 1a). Blood draws for the study were always combined with scheduled blood draws for clinical care. After collection, the samples were left to clot for 30 minutes to 2 hours, and centrifuged at 15000 *g* for 2 minutes at room temperature. Serum aliquots were stored at −80 °C, as described previously.^27^

Clinical data on neonatal characteristics were obtained during the study period from the electronic patient records. SGA was defined as birth weight below the 2.3 percentile according to the Dutch reference curve.^28^ Continuous non-normally distributed data as medians with interquartile range (IQR) and categorical data were expressed as counts with percentage (%). Patient characteristics were compared between infants born SGA versus infants born appropriate for gestational age (AGA) using a Mann-Whitney U test for continuous variables, and a Chi-square or Fisher’s exact test for categorical variables, depending on the distribution of the data.

### Healthy adults

We obtained serum samples from six healthy adults from Sanquin, Amsterdam, the Netherlands. Serum was obtained by leaving whole blood to clot for at least 30 minutes at room temperature and then centrifuged at 1800 g for 20 min at room temperature. Aliquots were stored at −80 °C until analysis. Ethical approval was obtained from the Sanquin Ethical Advisory Board.

### Serum sample preparation

Serum aliquots from the PRIDICT-BPD study cohort and healthy controls were thawed at room temperature (RT) and 10 μL was diluted 1:60 in 100 mM Tris(hydroxymethyl)aminomethane hydrochloride (Tris-HCl, Life Technologies, UK) (pH = 8.0). 9 μL diluted serum was mixed with 5 μL of 20 mM Tris(2-choloroethyl)phosphine (Thermo Fisher Scientific, Rockford, IL), 80 mM chloroacetamide (Sigma Aldrich, St Louis, MO) in 100 mM Tris-HCl (pH = 8.0). After incubation at 95 °C for 5 minutes, samples were cooled down to RT and proteins were digested overnight at 25 °C with 100 ng MS-grade Trypsin Gold (Promega, Madison, WI) in 30 μL of 50 mM Tris-HCl (pH = 8.0). Samples were acidified to a final concentration of 1% (v/v) trifluoroacetic acid (Thermo Fisher Scientific, Rockford, IL), 8x diluted in 0.1% formic acid in water (Biosolve, NL) and stored at −80 °C until MS analysis. Quality control (QC) samples were made by pooling equal aliquots of all samples in the cohort. 20 μL of the samples were loaded onto EvoTip Pure tips (EvoSep, Denmark) according to manufacturer’s guidelines.

### Mass spectrometry analysis

Samples were analyzed using an Evosep One liquid chromatography system (Evosep, Denmark)^29^ coupled to an Orbitrap Fusion™ Lumos™ Tribrid™ Mass Spectrometer (Thermo Fischer Scientific) equipped with an electrospray ionization source with a spray voltage of 2150 V. Peptides were separated using the predefined 30 samples / per day method on a 15 cm × 150 μm, 1.5 μm Performance Column (EV1137, EvoSep, Denmark) with 0.1% formic acid in water or acetonitrile (Biosolve, NLD) as mobile phase A and B, respectively. MS data were acquired with data independent acquisition mode. For MS1 a scan from 390−1010 m/z at 60 K resolution was made (100% normalized automatic gain control, target (AGC); 100 ms maximal injection time). Next, 75 consecutive MS2 scans were made with an 8 m/z window size and a 1 m/z overlap at 30 K resolution with a precursors mass range of 400−1000 at 300% AGC and 54 ms maximal injection. Higher-energy collisional dissociation fragmentation was set to normalized collision energy of 23%. All spectra were recorded in centroid mode and the default charge state was set to 3.

### Data processing

A spectral library was generated in DIA-NN (version 1.8.1)^30^ using single-pass mode as neural network classifier, protein inference based on the genes from the reviewed FASTA (20423 entries, downloaded on 08 August 2023), and robust LC (high accuracy) for the quantification strategy. Proteins were then identified in the samples using DIA-NN with the theoretical library and match-between-runs, but without heuristic protein inference and shared spectra. Data was analyzed using R (version 4.1.1)^31^ and tidyverse (version 1.3.2)^32^ for data processing. Protein group label-free quantitation (LFQ) values were kept only if intensities were based on two or more unique precursors (Table S3a). Data was log2 transformed and proteins were considered to be accurately quantified proteins if they were identified in at least 50% identification per time point and 40% per group (adults or infants) across the entire cohort (Table S3b). Imputation was performed using a normal distribution (downshift of 1.8, width of 0.3).

### Data analysis

Venn diagram was made with Eulerr^33^ and tissue enrichment was performed using TissueEnrich.^34^ Protein dynamics to theoretical shapes were investigated based on the squared Pearson correlation (similarity score) calculated with Hmisc^35^ based on the median protein intensity over infants per timepoint using the imputed data. The effect size over time was calculated as the area for every protein using Flux^36^ based on the median intensity per timepoint using the imputed data, in which the first timepoint was used to normalize to zero. Increasing and decreasing trends in protein abundances were classified based on a r-squared correlation ≥ 0.5 and absolute area (|effect size|) ≥ 20. Biological process enrichment analysis was performed using Metascape with all 1492 identified protein symbol names (Table S3a) as background and default settings. Briefly, terms with *P*-value < 0.05, a minimum count of 3 and an enrichment factor of more than 1.5 were grouped into clusters based on membership similarities (Kappa scores > 0.3).^37^ The most statistically significant term within a cluster was selected to represent the cluster, selecting only the top-20 terms. Cytoscape was used to visualize the networks.^38^ For exploration of the complement cascade, we manually annotated the proteins in the cascade based on literature and visualized their effect size.^39–42^ Statistical significance was determined using moderated t-tests using Limma^43^ using the imputed data. Correction for multiple testing was performed using the Benjamini-Hochberg method and a *P*-adjusted value < 0.05 and absolute log-fold change (|LFC|) of >1 was considered statistically significant and biologically relevant. Differences in longitudinal trajectories between groups was determined by subtracting the median coefficient of variation (CV) of the AGA group from the CV median of the SGA group for each protein. Differences in abundance were determined by calculating the LFC based on the median protein abundance in the AGA group compared to the SGA group. Differences in development were considered stable at a relative CV below 30%.

## Results

### Patient cohort and serum proteomics analysis

In the PRIDICT-BPD study, a total of 67 infants with a median GA of 27.6 weeks (IQR 26.6, 28.6 weeks) and a median birth weight of 1000 gram (IQR 810, 1190 gram) were included. Eighteen (26.9%) of these preterm infants were born SGA. The sample sets obtained at day 3 (*n* = 65, 97.0%), followed by day 7 (*n* = 56, 83.6%) and day 14 (*n* = 49, 73.1%) were the most complete, whereas for day 0 (*n* = 31, 46.3%) and 28 (*n* = 27, 40.3%) samples were less complete (Fig. 1a, b). From day 0 up to day 14, samples were missing mostly due to logistic reasons, however at day 28 samples were missing due to discharge from the NICU, as described in the primary publication on this cohort.^27^

To evaluate the robustness of our unbiased label-free MS-based proteomics workflow (Fig. 1a), we performed seven repeated measurement of a study wide pooled sample. Out of the 910 identified proteins in this pool, 874 proteins were reliably quantified with a coefficient of variation (CV) below 30% (Fig. 1c, Table S3c). This robustness is exemplified by the stability in protein levels of hemoglobin subunits, hemoglobin zeta (HBZ), epsilon (HBE), gamma-1 (HBG-1), gamma-2 (HBG-2), alpha (HBA), beta (HBB), delta (HBD) and mu (HBM) across repeated injections of the study wide pool (Fig. 1d).

### Global differences in the serum proteome between adults and preterm infants

To assess the circulating proteome of preterm infants, we explored variety and abundances of proteins in preterm infant (Table S1) compared to adult serum (Table S2). More proteins were quantified in preterm infants than in adults serum (Fig. S1a). This was due to increased quantification of proteins in the lower regions of the measurement range. Because a similar distribution is prerequisite for linear models,^43^ we opted to further compare the serum proteomes of preterm infants and adults solely qualitatively (Fig. S1b). A total of 515 proteins were present in both adults and preterm infant serum, while four proteins were only observed in adults (HBD, neutrophil elastase (ELANE), apolipoprotein C-IV (APOC4), and fructose-biphosphate aldolase B (ALDOB). In preterm infant serum we were able to identify 302 unique proteins, including known fetal and neonate-specific hemoglobulin subunits (HBZ, HBE, HBG-2, and HBM) as well as various collagen chains (Fig. S1c, Table S3d). Out of the 302 infant-specific proteins, 131 proteins were enriched for 23 different tissues. Most tissue specific proteins originated from the placenta (e.g. protein delta homolog 1, DLK1), bone marrow (e.g. porphobilinogen deaminase, HBMS) or cerebral cortex (e.g. protein kinase C-binding protein NELL2, NELL2) (Fig. S1d, e, Table S3e).

### Development of specific protein profiles with postnatal age

**Fig. 2:**
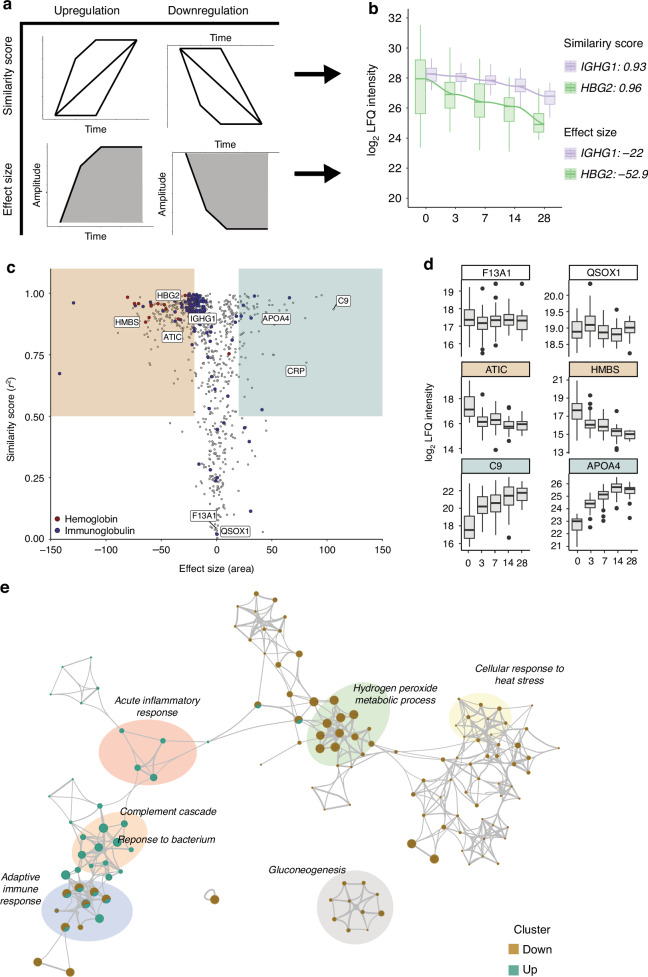
Classification of protein levels changing after birth in preterm infants. **a** Graphical visualisation of the approach to classifying proteins that show changes over time based on 1) similarity score (top left) calculated as the squared Pearson correlation to theoretical changes over time and 2) effect size (bottom left) calculated as the area of the protein level changes over time normalised to the protein levels at day 0 based on imputed data. **b** The label free quantification (LFQ)-intensity levels for immunoglobulin heavy gamma 1 (IGHG1, purple) and haemoglobin subunit gamma-2 (HBG2, green) per time point in infants with each sample shown using dots and the distribution with Tukey box-and-whisker plot. On the right the similarity score and effect size is depicted. **c** Similarity score plotted against the effect size for each protein represented as a dot. Red dots indicate haemoglobins and blue dots immunoglobulins, all other proteins are represented in grey dots. The squared in the plot show the threshold set for the classification of proteins for which the levels decrease (orange) or increase (green) with time after birth. **d** Tukey box-and-whisker plot representing the distribution of LFQ-intensity levels of coagulation factor XIIIa (F13A1), sulfhydryl oxidase 1 (QSOX1), bifunctional purine biosynthesis protein (ATIC), porphobilinogen deaminase (HMBS), complement component 9 (C9) and apolipoprotein A-IV (APOA4) per timepoints. Proteins panel backgrounds represent their classification. **e** Enrichment network visualization based on proteins classified as decreasing (orange) or increasing (green) with time with manual annotation of highlighted enrichment terms. Nodes represented each term within by pie charts indicating their associations with each classification.

To characterize protein changes associated to age-related changes after preterm birth, we classified proteins and their post-partum abundance trajectories based on their similarity to theoretical developmental (r^2^ > 0.50) trajectories over time. To distinguish proteins with similar developmental profiles with differences in magnitude compared to baseline (cord blood), exemplified for HGB2 and IGHG1 (Fig. 2b), we also accounted for effect sizes (|area|>20) (Fig. 2a, Table S3f). In this way, this approach can identify the similar trend over time between these proteins as well as the differences in protein level decrease.

Overall, 507 proteins, including coagulation factor XIIIa (F13A1, area: −0.09; r^2^: 0.15) and sulfhydryl oxidase 1 (QSOX1, area: 0.7; r^2^: 0.01), did not change in a clear age-dependent pattern over time (Fig. 2c, white area – 2D). Out of the 315 protein levels that were classified as changing with time postnatally, the majority of the proteins (235) levels decreased in abundances over time (Fig. 2c, orange box). In line with literature, we observed decreasing protein levels for embryonic and fetal hemoglobin (HBG1, HBG2, HBZ, HBM, HBE1) and IgG (IGHG1, IGHG2, IGHG3, IGHG4) with postnatal age (Fig. S2, S3). Additionally, we observed non-described developmental decreases in protein abundances e.g. for bifunctional purine biosynthesis protein ATIC (ATIC, area: −49.3; r^2^: 0.97) and porphobilinogen deaminase (HMBS, area: −71.7; r^2^: 0.93) (Fig. 2d). Proteins with decreased levels associated with gluconeogenesis, cellular response to heat stress, and hydrogen peroxide metabolic process (Fig. 2e, S4, Table S3g, S3h). The 79 proteins with increasing abundance postnatally (Fig. 2c, green box) included CRP (area: 81.3; r^2^: 0.67), complement 9 (C9, area: 105.8; r^2^: 0.97) and apolipoprotein A-IV (APOA4, area: 63.4; r^2^: 0.93) (Fig. 2d). Proteins with increased abundances were associated with acute inflammatory response, complement cascade and response to bacterium (Fig. 2e, S3, Table S3g, S3h). Notably, both increasing and decreasing protein levels with postnatal age were observed for proteins involved in the adaptive immune system and selenium micronutrient network (Fig. 2e, S3).

**Fig. 3:**
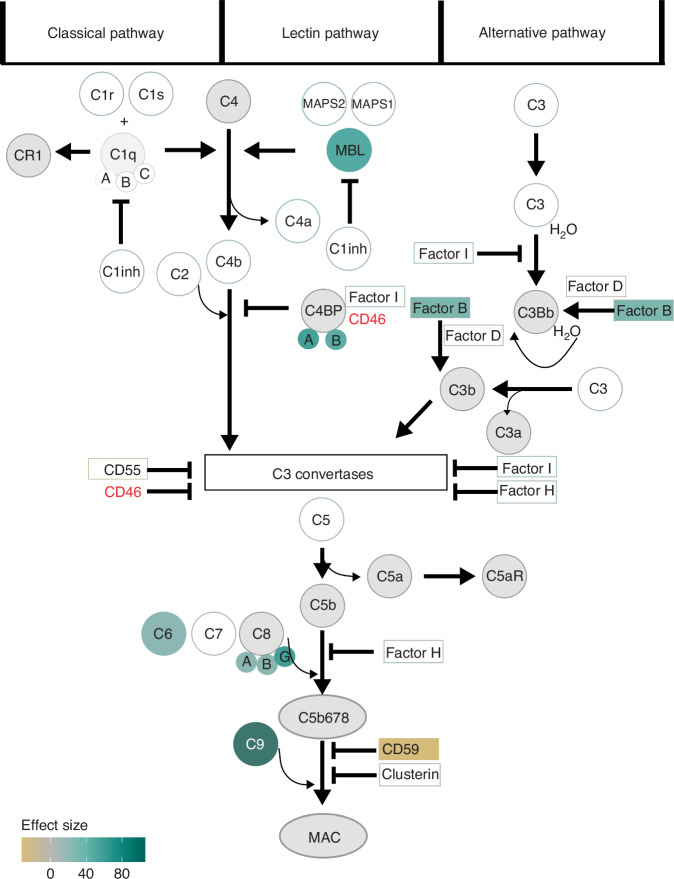
Schematic representation of changes in complement cascade proteins. Graphical representation of the complement cascade annotated by the effect size as calculated in Fig. 3, and left white if it was classified as stable over time. Red letters indicate proteins were not quantified in this study and grey circles indicate changes in protein levels the mass spectrometer is not capable of distinguishing. Square boxes represent complement cascade inhibitors.

The postnatal development of the complement system in the first four weeks in preterm infants remains not completely elucidated, yet C9 was the most extreme increasing postnatal change (area > 100) (Fig. 2c). Therefore, we investigated the changes in levels of proteins involved in the entire complement cascade. In total, we quantified 36 proteins covering the entire complement cascade (Fig. 3, S5). The majority of the complement cascade regulating proteins remained stable (|area|< 20, white box) in abundances over time, including CD55 (area: −12.1; r^2^: 0.90), clusterin (area: −10; r^2^: 0.74), complement factor I (area: 17.2; r^2^: 0.97) complement factor D (area: −1.8; r^2^: 0.88) and complement factor H (area: 11.6; r^2^: 0.94). However, decreasing levels of CD59 (area: −31.6; r^2^: 0.93) and increasing levels of complement factor B (area: 28.5; r^2^: 0.98) were observed. Especially proteins that are a part of the membrane attack complex, complement component 6 (area: 20.9; r^2^: 0.80), 7 (area: 8.1; r^2^: 0.41), and 8 (alpha, C8A, area: 22.1; r^2^: 0.92; beta, C8B, area: 22.0; r^2^: 0.90; gamma, C8G, area: 56.8; r^2^: 0.93), increased in abundance with time postnatally.

### Development of specific protein profiles associated with SGA

Because SGA is a known risk factor in preterm infants for short- and long-term health impairment, we investigated protein differences between preterm neonates born SGA with preterm neonates born AGA in protein profiles at birth. In this study, SGA infants were born with a median gestational age of 27.9 weeks (IQR 27.1, 28.9) and a median birth weight of 810 gram (IQR 590, 919 gram). As expected, SGA infants had a significantly lower birth weight, were significantly more often born via caesarean section, more often diagnosed with moderate or severe BPD and were more often on invasive mechanical ventilation compared with infants born AGA (Table S1)

**Fig. 4:**
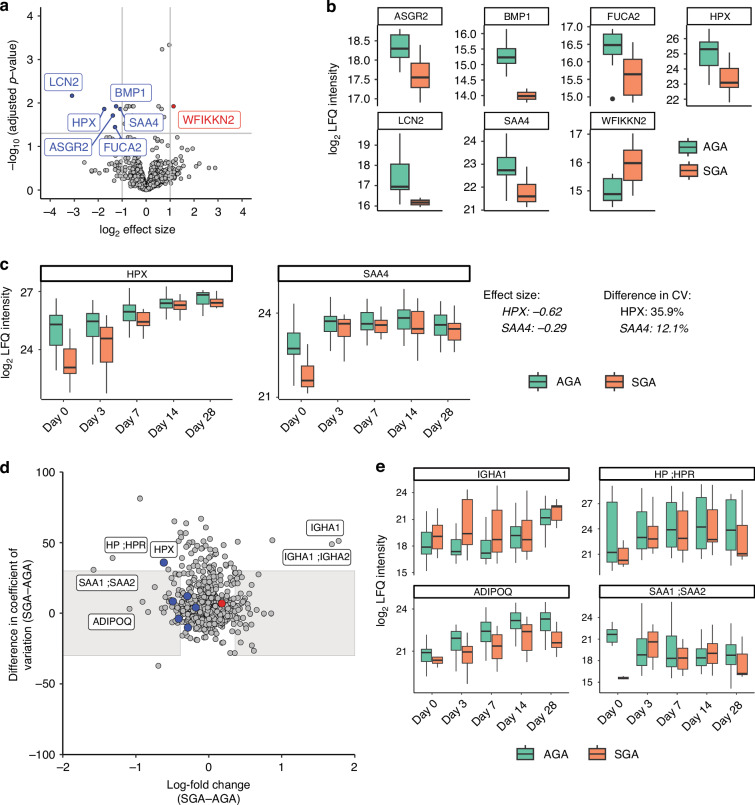
Differences in the serum proteome of SGA and AGA infants. **a** Volcano plot representing the comparison of differences in cord blood samples from small for gestational age (SGA) infants (*n* = 10) to appropriate for gestational age (AGA) infants (*n* = 21) determined with imputed data, with significantly up-regulated proteins in red and down-regulated proteins in blue. The x-axis represents the log_2_ fold-change in protein levels and the y-axis represents the -log_10_ t-test *p*-value after Benjamini-Hochberg correction. **b** Tukey box-and-whisker plot representing the distribution of label-free quantification (LFQ)-intensity levels of all significant proteins in SGA (orange) and AGA (green) infants, including hemopexin (HPX), asialoglycoprotein receptor 2 (ASGR2), bone morphogenetic protein 1 (BMP1), serum amyloid A-4 protein (SAA4), plasma alpha-L-fucosidase (FUCA2), neutrophil gelatinase-associated lipocalin (LCN2) and WAP, Kazal, immunoglobulin, Kunitz and NTR domain-containing protein 2 (WFIKKN2). **c** Tukey box-and-whisker plot representing the distribution of LFQ-intensity levels in SGA (orange) and AGA (green) infants of HPX and SAA4 with the difference in CV and LFQ (effect size) over time on the right. **d** Differences in trajectories over time after birth. The x-axis represents the difference in abundance (log-fold change) and the y-axis represent the difference in trend over time. The black square represents all proteins within the normal distribution (0.05 ≤ value ≤ 0.95). Significantly up-regulated proteins in cord blood of SGA infants are highlighted in red and down-regulated proteins in blue. **e** Tukey box-and-whisker plot representing the distribution of LFQ-intensity levels in SGA (orange) and AGA (green) infants of adiponectin (ADIPOQ), haptoglobin (HP; HPR), serum amyloid A-1/2 (SAA1; SAA2) and immunoglobulin heavy constant alpha 1 (IGHA1).

Comparison of the sera at birth of AGA versus SGA preterm infants revealed limited differences, with seven proteins significantly altered (Fig. 4a, Table S3i). The abundances of six of these proteins were reduced in SGA infants, including hemopexin (HPX), asialoglycoprotein receptor 2 (ASGR2), bone morphogenetic protein 1 (BMP1), plasma alpha-L-fucosidase (FUCA2), serum amyloid A-4 protein (SAA4) and neutrophil gelatinase-associated lipocalin (LCN2). Only WAP, Kazal, immunoglobulin, Kunitz NTR domain-containing protein 2 (WFIKKN2) exhibited higher levels in SGA preterm infants (Fig. 4b). No enrichment for biological processes was observed. To explore whether these significant proteins remained different over time, the protein levels over time were plotted. The levels for most proteins, e.g. SAA4, normalized rapidly. However the levels of HPX, a glycoprotein that binds free heme in circulation to prevent iron loss and iron-induced oxidative damage, normalized slowly over time (Fig. 4c, S6), with a more rapid increase in levels for SGA infants.

To further investigate differences in developmental trajectories between SGA and AGA preterm infants, we plotted the difference in developmental trends (longitudinal CV, |CV|> 30%) in comparison to the difference in protein levels across all timepoints (LFC < Q_0.5_ or LFQ > Q_0.95_) (Table S3j). As expected based on Fig. 5c, HPX was observed with a difference in developmental trend and an effect size outside the range of normal distribution (grey box). This showed this approach can be used to identify deviations in postnatal trends (Fig. 4c, d). The most pronounces differences (|LFC|> 1) included the upregulation of immunoglobulin heavy constant alpha 1 (IGHA1) and downregulation of haptoglobin (HP) in SGA infants compared to AGA infants (Fig. 4e). In total, 69 proteins showed similar trends (|CV|< 30%) with different protein levels. Enrichment analysis revealed 24 proteins enriched for response to bacteria and the immune system, including C4b-binding protein alpha chain (C4BPA), -beta chain (C4BPB), leucine-rich alpha-2-glycoprotein (LRG1), IGHG2;IGHG4 and IGHG1;IGHG3;IGHG4 (Table S3k). Finally, the most extreme differences in protein levels with similar trends over time were the lower levels of the insulin sensitizing hormone adiponectin (ADIPOQ) and acute phase protein serum amyloid A (SAA)-1/2 in SGA infants compared to AGA infants (Fig. 4e).

## Discussion

We highlight the potential of MS-based serum proteomics in pediatric research by studying the proteome of preterm infants, using residual material from a clinical cohort (*n* = 67).^27^ This is especially powerful in this vulnerable population due to the limited blood volume required^15^ and information-rich output it provides. We showcase this technique to be able to capture changes in the proteomic landscape associated to postnatal changes in protein levels and identify differences in proteins trajectories according to the degree of fetal growth restriction (SGA compared to AGA).

In line with our findings, others have previously reported differences in protein levels of fetal hemoglobin, alpha-fetoprotein, alpha-2-macroglobulin, haptoglobin and collagen-alpha chains between adults and full-term infants.^25,44^ Strikingly, the proteomic depth measured by MS-based serum proteomics of preterm infants is much deeper compared to healthy adults, with a substantial group of proteins unique to infants and a difference in data distribution. To our knowledge, this has not been described previously. However, it has been shown that for albumin the concentration is drastically lower in preterm infants (20 g/L albumin, 35 μg/μL protein)^45,46^ compared to later in life (34–54 g/L albumin, 70 μg/μL protein).^47,48^ We hypothesize that the increased proteomic depth is in part also an effect of the differences in total plasma protein and albumin concentrations in infants and adults, and that through albumin depletion some of the infants-specific proteins may be identified in adults as well. However we expect other proteins such as the placenta enriched proteins to remain infant-specific. Similarity we expect some cerebral cortex protein to remain specific, since the cerebral cortex undergoes an important developmental period occurring between 24 to 40 weeks postmenstrual age, including cerebral maturation.^49^ As these infants are born during this time the presence of cerebral-cortex enriched proteins in the blood may indicate immaturity of the blood–brain barrier in preterm infants. This is in accordance with previous studies that showed increased levels of proteins linked to neurodevelopment in cerebral spinal fluid in preterm infants compared to full term infants.^50^

To characterize changes in protein abundance levels of circulating proteins over time after birth, we utilized the dynamics of well-known developmental proteins as a reference, including embryonic- (HBZ, HBE), fetal hemoglobin (HBG, HBA), and immunoglobulins. For hemoglobin, the switch from fetal to adult hemoglobin (HBA, HBB, or HBD) starts shortly after birth,^17^ and is completed after approximately six months.^51^ In consonance with previous literature,^17,18,51^ we observed decreasing protein levels for embryonic (HBZ, HBE) and fetal (HBG, HBA) hemoglobin over time after birth in preterm infants. Furthermore, in full-term infants maternal IgG levels decrease postnatally and infant IgG gradually increases, resulting in a net decrease of total IgG in the first six months,^20,21^ which was also observed for preterm infants. Furthermore, C9 showed the largest increase in protein levels over time. Short-term developmental profiles of the complement system have previously been described for the first week after birth in a cohort of full-term infants.^16^ Through evaluation of the protein level changes of this entire complement cascade we observed that drastic changes occurred in most proteins of the MAC complex, which consists of C5b, C6, C7, C8α, C8β, C8γ and multiple copies of C9.^52^ Notably, C7 remained stable over time in preterm infants, in contrast to the decreasing levels observed for full-term infants.^16^ Often, these soluble forms of the MAC-complex are detected in the serum of patients suffering from infections,^53–55^ including septic patients.^56^ Previous studies have shown that infants have lower concentrations of C9 at birth compared to adults.^57^ Moreover, C9 has been shown to play an important role in killing of the Escherichia coli bacteria in septic infants,^58^ indicating the importance of C9 in sepsis. In this cohort 27 (40.3%) infants had culture-proven sepsis. It may be that the drastic increase in C9 protein levels over time is, at least partially, due to the immune response of septic infants in this cohort (Table S1). For further characterization of the effect of sepsis on this cascade, it may be interesting to look for differences between septic and postnatally age-matched non-septic infants at the time surrounding a sepsis episode and include measurement of C5b, as this initiates the formation of MAC resulting in cell lysis and inflammatory triggers,^59^ or other complement activation products.

The total number of proteins that were classified to be changing over time after preterm birth in this analysis underlines that these preterm infants indeed undergo drastic changes in circulating protein levels after birth, which should be accounted for in the characterization of disease-related changes. To further explore this, we evaluated differences in cord blood and age-related changes after birth for SGA, a known risk factor in preterm infants for short- and long-term health impairment. In cord blood, which is often used to study preterm infants, we found few significant differences in protein levels between SGA infants compared to AGA infants. Moreover, the significantly different protein levels between SGA infants and AGA infants were not linked to a specific biological process. In contrast, longitudinal monitoring of the groups allowed for the identification of protein differences associated with SGA infants. The observation of ADIPOQ indicates the validity of this approach as it is one of the most important hormones in insulin sensitivity and homeostasis energy,^60^ and has been described to be found at lower levels in cord blood of SGA infants compared with AGA infants.^61^ This is associated with an altered pattern of fat accumulation in this population.^62^ Among the other proteins with similar developmental trends and different protein levels, we also found lower levels of platelet proteins including platelet basic protein (PPBP) and platelet factor 4 (PF4). This is likely associated with the more frequent occurrence of thrombocytopenia in SGA infants.^63^ Of note, higher levels of ADIPOQ, as seen in AGA infants, have been suggested to be associated with megakaryocytic maturation in mice,^64,65^ which is responsible for the release of platelets from the bone marrow. Thus the lower levels of platelet proteins, which are known to correlate to platelet count,^66^ may be a result of the lower levels of ADIPOQ. Besides ADIPOQ and platelets, we observed alterations in protein levels involved in the adaptive (IGHG1;IGHG3;IGHG4) and innate C4BPA, C4BPB, C8G) immune system. As IgG mediates the activation of complement to ward off infections, the higher levels of these proteins might elude to an ongoing infection. In addition, we also observed various proteins with a difference in both developmental trends and proteins levels (SAA1, HP, IGHA1, HPX and GLRX). The difference in protein abundances combined with increased variability in SGA infants could indicate that these proteins were also impacted by short-term complications, as SGA is a known risk-factor for complications, such as BPD (Table S1). This highlights the opportunity that MS-based proteomics entails to further study protein alterations in common complications including bleeding, BPD, and sepsis.

Our study has limitations that should be taken into consideration when interpreting our findings. Importantly, the limited differences between SGA and AGA infants at birth could also be a result of variation in the protein levels due to GA, lack of cord blood samples, and heterogeneity in the SGA diagnosis.^67–69^ SGA, especially in preterm infants < 32 weeks GA, is commonly used as a proxy for intrauterine growth restriction.^70^ However they are not interchangeable,^69^ as the definition of SGA does not distinguish between neonates who are small but otherwise healthy and those with growth restriction due to pathological conditions as one would see in intrauterine growth restriction.^71,72^ Moreover, the definition of SGA used here is based on a set threshold according to the Dutch reference curve,^28^ and is thus used categorically. Some protein levels may, however, be dependent upon the continuous scale of birthweight instead, which may induce variation and thus limit statistical power in the comparison. Secondly, MS-based proteomics as applied in this study is not capable of distinguishing between the activated and non-activated form of proteins such as for complement component 5 and its cleaved C5a counterpart. Therefore, we have to be careful in drawing conclusions on the changes in the activation of elements in the complement cascade that preterm infants undergo. Further exploration could also be performed with MS using different acquisition approaches as it could then be capable of evaluating cleavage-specific changes in proteins across the entire complement cascade. Another limitation that needs to be considered is the relatively low number of infants included in the study. Given the explorative nature of our study, we did not perform a power analysis beforehand.

## Conclusions

This work demonstrates the potential of unbiased MS-based serum profiling in a vulnerable population of preterm infants with low blood volumes. This approach allowed for the evaluation of systematic changes in development after preterm birth through characterization of biological processes in which these circulating proteins are involved and further characterization of the complement cascade, one of the major processes in the human body. We also demonstrate that longitudinal monitoring of this population with this approach can provide insight into perturbations associated with SGA, and can thus be an important tool to study disease development and progression in preterm infants. These findings provide a stepping stone to focus on health complications that are frequently encountered in preterm infants, along with long-term health consequences.

## Supplementary information

‏‏‏‏Supplemental Figures and Tables

Supplemental Table3

## Data Availability

The datasets generated and/or analysed during the current study are available from the corresponding author on reasonable request.

## References

[1] Ohuma, E. O. et al. National, regional, and global estimates of preterm birth in 2020, with trends from 2010: a systematic analysis. *Lancet* **402**, 1261–1271 (2023). 37805217 10.1016/S0140-6736(23)00878-4

[2] Walani, S. R. Global burden of preterm birth. *Int. J. Gynecol. Obstet.* **150**, 31–33 (2020). 10.1002/ijgo.13195 32524596

[3] Perin, J. et al. Global, regional, and national causes of under-5 mortality in 2000–19: an updated systematic analysis with implications for the Sustainable Development Goals. *Lancet Child Adolesc. Heal.* **6**, 106–115 (2022). 10.1016/S2352-4642(21)00311-4 PMC8786667 34800370

[4] Stoll, B. J. et al. Neurodevelopmental and growth impairment among extremely low-birth-weight infants with neonatal infection. *JAMA* **292**, 2357–2365 (2004). 15547163 10.1001/jama.292.19.2357

[5] Patel, R. M. Short- and long-term outcomes for extremely preterm infants. *Am. J. Perinatol.* **33**, 318–328 (2016). 26799967 10.1055/s-0035-1571202 PMC4760862

[6] Barfield, W. D. Public health implications of very preterm birth. *Clin. Perinatol.* **45**, 565–577 (2018). 30144856 10.1016/j.clp.2018.05.007 PMC6193262

[7] Mwaniki, M. K., Atieno, M., Lawn, J. E. & Newton, C. R. J. C. Long-term neurodevelopmental outcomes after intrauterine and neonatal insults: a systematic review. *Lancet* **379**, 445–452 (2012). 22244654 10.1016/S0140-6736(11)61577-8 PMC3273721

[8] Grisaru-Granovsky, S. et al. Mortality and morbidity in preterm small-for-gestational-age infants: a population-based study. *Am. J. Obstet. Gynecol.* **206**, 150.e1–7 (2012). 21982023 10.1016/j.ajog.2011.08.025

[9] Muhihi, A. et al. Risk factors for small-for-gestational-age and preterm births among 19,269 Tanzanian newborns. *BMC Pregnancy Childbirth* **16**, 110 (2016). 27183837 10.1186/s12884-016-0900-5 PMC4869183

[10] Sharma, D., Shastri, S. & Sharma, P. Intrauterine growth restriction: antenatal and postnatal aspects. *Clin. Med. Insights Pediatr.* **10**, 67–83 (2016). 27441006 10.4137/CMPed.S40070 PMC4946587

[11] Løhaugen, G. C. C. et al. Small for gestational age and intrauterine growth restriction decreases cognitive function in young adults. *J. Pediatr.* **163**, 447–453.e1 (2013). 23453550 10.1016/j.jpeds.2013.01.060

[12] Cai, S., Thompson, D. K., Anderson, P. J. & Yang, J. Y.-M. Short- and long-term neurodevelopmental outcomes of very preterm infants with neonatal sepsis: a systematic review and meta-analysis. *Children* **6**, 131 (2019). 10.3390/children6120131 PMC6956113 31805647

[13] Aladangady, N., McHugh, S., Aitchison, T. C., Wardrop, C. A. J. & Holland, B. M. Infants’ blood volume in a controlled trial of placental transfusion at preterm delivery. *Pediatrics* **117**, 93–98 (2006). 16396865 10.1542/peds.2004-1773

[14] Persad, E. et al. Interventions to minimize blood loss in very preterm infants-A systematic review and meta-analysis. *PLoS One* **16**, e0246353 (2021). 33556082 10.1371/journal.pone.0246353 PMC7870155

[15] Geyer, P. E. et al. Plasma proteome profiling to assess human health and disease. *Cell Syst.* **2**, 185–195 (2016). 27135364 10.1016/j.cels.2016.02.015

[16] Bennike, T. B. et al. Preparing for Life: Plasma proteome changes and immune system development during the first week of human life. *Front. Immunol*. **11**, 578505 (2020). 10.3389/fimmu.2020.578505 PMC7732455 33329546

[17] Sankaran, V. G. & Orkin, S. H. The switch from fetal to adult hemoglobin. *Cold Spring Harb. Perspect. Med.* **3**, a011643 (2013). 23209159 10.1101/cshperspect.a011643 PMC3530042

[18] Bednarczuk, N., Williams, E. E., Kaltsogianni, O., Greenough, A. & Dassios, T. Postnatal temporal changes of foetal haemoglobin in prematurely born infants. *Acta Paediatr.* **111**, 1338–1340 (2022). 35429186 10.1111/apa.16360 PMC9321545

[19] Grether, J. K. et al. Prenatal and newborn immunoglobulin levels from mother-child pairs and risk of autism spectrum disorders. *Front. Neurosci.* **10**, 218 (2016). 27242422 10.3389/fnins.2016.00218 PMC4870252

[20] Ballow, M., Cates, K. L., Rowe, J. C., Goetz, C. & Desbonnet, C. Development of the immune system in very low birth weight (less than 1500 g) premature infants: concentrations of plasma immunoglobulins and patterns of infections. *Pediatr. Res.* **20**, 899–904 (1986). 3748663 10.1203/00006450-198609000-00019

[21] Berg, T. Immunoglobulin levels in infants with low birth weights. *Acta Paediatr.* **57**, 369–376 (1968). 10.1111/j.1651-2227.1968.tb07307.x 4178846

[22] Suski, M. et al. Prospective plasma proteome changes in preterm infants with different gestational ages. *Pediatr. Res*. **84**, 104–111 (2018). 10.1038/s41390-018-0003-2 29795197

[23] Suski, M. et al. Plasma proteome changes in cord blood samples from preterm infants. *J. Perinatol*. **38**, 1182–1189 (2018). 10.1038/s41372-018-0150-7 29910464

[24] Zasada, M. et al. Comparative two time-point proteome analysis of the plasma from preterm infants with and without bronchopulmonary dysplasia. *Ital. J. Pediatr*. **45**, 112 (2019). 10.1186/s13052-019-0676-0 PMC6708124 31445514

[25] McCafferty, C. et al. Plasma proteomic analysis reveals age-specific changes in platelet- and endothelial cell–derived proteins and regulators of plasma coagulation and fibrinolysis. *J. Pediatr.* **221**, S29–S36 (2020). 10.1016/j.jpeds.2020.01.051 32482231

[26] Zhong, W. et al. The development of blood protein profiles in extremely preterm infants follows a stereotypic evolution pattern. *Commun. Med.* **3**, 107 (2023). 37532738 10.1038/s43856-023-00338-1 PMC10397184

[27] Romijn, M. et al. Glucocorticoid signature of preterm infants developing bronchopulmonary dysplasia. *Pediatr. Res.* **94**, 1804–1809 (2023). 37355738 10.1038/s41390-023-02690-3

[28] Hoftiezer, L. et al. From population reference to national standard: new and improved birthweight charts. *Am. J. Obstet. Gynecol.* **220**, 383.e1–383.e17 (2019). 30576661 10.1016/j.ajog.2018.12.023

[29] Bache, N. et al. A Novel LC system embeds analytes in pre-formed gradients for rapid, ultra-robust proteomics. *Mol. Cell. Proteom.* **17**, 2284–2296 (2018). 10.1074/mcp.TIR118.000853 PMC6210218 30104208

[30] Demichev, V., Messner, C. B., Vernardis, S. I., Lilley, K. S. & Ralser, M. DIA-NN: neural networks and interference correction enable deep proteome coverage in high throughput. *Nat. Methods* **17**, 41–44 (2020). 31768060 10.1038/s41592-019-0638-x PMC6949130

[31] R Core Team. R: A Language and Environment for Statistical Computing. https://www.R-project.org/ (R Foundation for Statistical Computing, Vienna, Austria, 2022).

[32] Wickham, H. et al. Welcome to the Tidyverse Tidyverse package. *JOSS*, **4**, 1686 (2019).

[33] Larsson, J. eulerr: Area-Proportional Euler and Venn Diagrams with Ellipses. R Package https://cran.r-project.org/package=eulerr (2022).

[34] Jain, A. & Tuteja, G. TissueEnrich: Tissue-specific gene enrichment analysis. *Bioinformatics* **35**, 1966–1967 (2019). 30346488 10.1093/bioinformatics/bty890 PMC6546155

[35] Harrell, F. E. J. Hmisc: Harrell Miscellaneous. R Package https://CRAN.R-project.org/package=Hmisc (2023).

[36] Jurasinski, G., Koebsch, F., Guenther, A. & Beetz, S. flux: Flux rate calculation from dynamic closed chamber measurements. R Package https://CRAN.R-project.org/package=flux (2022).

[37] Zhou, Y. et al. Metascape provides a biologist-oriented resource for the analysis of systems-level datasets. *Nat. Commun.* **10**, 1523 (2019). 30944313 10.1038/s41467-019-09234-6 PMC6447622

[38] Shannon, P. et al. Cytoscape: a software environment for integrated models of biomolecular interaction networks. *Genome Res.* **13**, 2498–2504 (2003). 14597658 10.1101/gr.1239303 PMC403769

[39] Shinjyo, N., Kagaya, W. & Pekna, M. Interaction between the complement system and infectious agents – a potential mechanistic link to neurodegeneration and dementia. *Front. Cell. Neurosci*. **15**, 710390 (2021). 10.3389/fncel.2021.710390 PMC8365172 34408631

[40] Pierik, E. et al. Dysregulation of complement activation and placental dysfunction: a potential target to treat Preeclampsia? *Front. Immunol.* **15**, 3098 (2020). 10.3389/fimmu.2019.03098 PMC6974484 32010144

[41] Ng, N. & Powell, C. A. Targeting the complement cascade in the pathophysiology of COVID-19 disease. *J. Clin. Med.* **10**, 2188 (2021). 34069355 10.3390/jcm10102188 PMC8158769

[42] Girardi, G., Lingo, J. J., Fleming, S. D. & Regal, J. F. Essential role of complement in pregnancy: from implantation to parturition and beyond. *Front. Immunol.* **11**, 1681 (2020). 32849586 10.3389/fimmu.2020.01681 PMC7411130

[43] Ritchie, M. E. et al. limma powers differential expression analyses for RNA-sequencing and microarray studies. *Nucleic Acids Res.* **43**, e47–e47 (2015). 25605792 10.1093/nar/gkv007 PMC4402510

[44] Bjelosevic, S. et al. Quantitative age-specific variability of plasma proteins in healthy neonates, children and adults. *Mol. Cell. Proteom.* **16**, 924–935 (2017). 10.1074/mcp.M116.066720 PMC5417830 28336724

[45] Reading, R. F., Ellisb, R., Fleetwoodb, A., Ellis, R. & Fleetwood, A. Plasma albumin and total protein in preterm babies from birth to eight weeks. *Early Hum. Dev.* **22**, 81–87 (1990). 2364907 10.1016/0378-3782(90)90082-t

[46] Cartlidge, P. H. & Rutter, N. Serum albumin concentrations and oedema in the newborn. *Arch. Dis. Child.* **61**, 657–660 (1986). 3740904 10.1136/adc.61.7.657 PMC1777869

[47] Geyer, P. E., Holdt, L. M., Teupser, D. & Mann, M. Revisiting biomarker discovery by plasma proteomics. *Mol. Syst. Biol*. **13**, 942 (2017). 10.15252/msb.20156297 PMC5615924 28951502

[48] Weaving, G., Batstone, G. F. & Jones, R. G. Age and sex variation in serum albumin concentration: an observational study. *Ann. Clin. Biochem.* **53**, 106–111 (2016). 26071488 10.1177/0004563215593561

[49] Perrone, S. et al. Brain damage in preterm and full-term neonates: serum biomarkers for the early diagnosis and intervention. *Antioxidants* **12**, 309 (2023). 36829868 10.3390/antiox12020309 PMC9952571

[50] Leifsdottir, K. et al. The cerebrospinal fluid proteome of preterm infants predicts neurodevelopmental outcome. *Front. Pediatr*. **10**, 921444 (2022). 10.3389/fped.2022.921444 PMC9343678 35928685

[51] Wang, X. & Thein, S. L. Switching from fetal to adult hemoglobin. *Nat. Genet.* **50**, 478–480 (2018). 29610477 10.1038/s41588-018-0094-z PMC6419756

[52] Dudkina, N. V. et al. Structure of the poly-C9 component of the complement membrane attack complex. *Nat. Commun.* **7**, 10588 (2016). 26841934 10.1038/ncomms10588 PMC4742998

[53] Mook-Kanamori, B. B., Brouwer, M. C., Geldhoff, M., Ende, Avander & van de Beek, D. Cerebrospinal fluid complement activation in patients with pneumococcal and meningococcal meningitis. *J. Infect.* **68**, 542–547 (2014). 24412248 10.1016/j.jinf.2013.12.016

[54] Doorduijn, D. J. et al. Soluble MAC is primarily released from MAC-resistant bacteria that potently convert complement component C5. *Elife* **11**, e77503 (2022). 35947526 10.7554/eLife.77503 PMC9402229

[55] Westra, D. et al. Serological and genetic complement alterations in infection-induced and complement-mediated hemolytic uremic syndrome. *Pediatr. Nephrol.* **32**, 297–309 (2017). 27718086 10.1007/s00467-016-3496-0 PMC5203860

[56] de Nooijer, A. H. et al. Complement activation in severely ill patients with sepsis: no relationship with inflammation and disease severity. *Crit. Care* **27**, 63 (2023). 36797757 10.1186/s13054-023-04344-6 PMC9933299

[57] Lassiter, H. A., Watson, S. W., Seifring, M. L. & Tanner, J. E. Complement factor 9 deficiency in serum of human neonates. *J. Infect. Dis.* **166**, 53–57 (1992). 1607708 10.1093/infdis/166.1.53

[58] Lassiter, H. A., Wilson, J. L., Feldhoff, R. C., Hoffpauir, J. M. & Klueber, K. M. Supplemental complement component C9 enhances the capacity of neonatal serum to kill multiple isolates of pathogenic Escherichia coli. *Pediatr. Res.* **35**, 389–396 (1994). 8047374

[59] Horiuchi, T. & Tsukamoto, H. Complement-targeted therapy: development of C5- and C5a-targeted inhibition. *Inflamm. Regen.* **36**, 11 (2016). 29259684 10.1186/s41232-016-0013-6 PMC5725830

[60] Pittas, A. G., Joseph, N. A. & Greenberg, A. S. Adipocytokines and insulin resistance. *J. Clin. Endocrinol. Metab.* **89**, 447–452 (2004). 14764746 10.1210/jc.2003-031005

[61] Kamoda, T., Saitoh, H., Saito, M., Sugiura, M. & Matsui, A. Serum Adiponectin concentrations in newborn infants in early postnatal life. *Pediatr. Res.* **56**, 690–693 (2004). 15371566 10.1203/01.PDR.0000142711.24999.8A

[62] Ratnasingham, A., Eiby, Y. A., Dekker Nitert, M., Donovan, T. & Lingwood, B. E. Is rapid fat accumulation in early life associated with adverse later health outcomes? *Placenta* **54**, 125–130 (2017). 28104278 10.1016/j.placenta.2017.01.101

[63] Christensen, R. D. et al. Thrombocytopenia in small-for-gestational-age infants. *Pediatrics* **136**, e361–e370 (2015). 26216323 10.1542/peds.2014-4182 PMC4906543

[64] Malara, A. et al. The secret life of a megakaryocyte: emerging roles in bone marrow homeostasis control. *Cell. Mol. Life Sci.* **72**, 1517–1536 (2015). 25572292 10.1007/s00018-014-1813-y PMC4369169

[65] Fiaschi, T. et al. Globular Adiponectin as a complete mesoangioblast regulator: role in proliferation, survival, motility, and skeletal muscle differentiation. *Mol. Biol. Cell* **21**, 848–859 (2010). 20089845 10.1091/mbc.E09-04-0310 PMC2836966

[66] Geyer, P. E. et al. Plasma Proteome Profiling to detect and avoid sample-related biases in biomarker studies. *EMBO Mol. Med.* **11**, e10427 (2019). 31566909 10.15252/emmm.201910427 PMC6835559

[67] Physical status: the use and interpretation of anthropometry. Report of a WHO Expert Committee. *World Health Organization Technical Report Series* **854**, 1–452 (1995). 8594834

[68] Schlaudecker, E. P. et al. Small for gestational age: Case definition & guidelines for data collection, analysis, and presentation of maternal immunisation safety data. *Vaccine* **35**, 6518–6528 (2017). 29150057 10.1016/j.vaccine.2017.01.040 PMC5710996

[69] Finken, M. J. J. et al. Children born small for gestational age: differential diagnosis, molecular genetic evaluation, and implications. *Endocr. Rev.* **39**, 851–894 (2018). 29982551 10.1210/er.2018-00083

[70] Vayssière, C. et al. Fetal growth restriction and intra-uterine growth restriction: guidelines for clinical practice from the French College of Gynaecologists and Obstetricians. *Eur. J. Obstet. Gynecol. Reprod. Biol.* **193**, 10–18 (2015). 26207980 10.1016/j.ejogrb.2015.06.021

[71] Carducci, B. & Bhutta, Z. A. Care of the growth-restricted newborn. *Best. Pract. Res. Clin. Obstet. Gynaecol.* **49**, 103–116 (2018). 29571821 10.1016/j.bpobgyn.2018.02.003

[72] Gordijn, S. J., Beune, I. M. & Ganzevoort, W. Building consensus and standards in fetal growth restriction studies. *Best. Pract. Res. Clin. Obstet. Gynaecol.* **49**, 117–126 (2018). 29576470 10.1016/j.bpobgyn.2018.02.002

