# Supplementary material for: Distinct protein patterns related to postnatal development in small for gestational age preterm infants
Source: Pediatr Res. 2024 Aug 16;97(5):1722–31. doi: 10.1038/s41390-024-03481-0 (PMC12119372; doi:10.1038/s41390-024-03481-0)
Supplement: Supplementary file 1 — ‏‏‏‏Supplemental Figures and Tables [file 41390_2024_3481_MOESM1_ESM.pdf]

Patient characteristic	N=67	SGA (n = 18)	AGA (n = 49)
Gestational age, wk, median (IQR)	27.6 (26.6, 28.6)	27.9 (27.1, 28.9)	27.3 (26.4, 28.5)
Birth weight, g, median (IQR)	1000 (810, 1190)	810 (590, 919)	1100 (913, 1253)*
Small for gestational age, No. (%)	18 (26.9)		
Male sex, No. (%)	35 (52.2)	10 (55.6)	25 (51.0)
Singleton, No. (%)	47 (70.1)	14 (77.8)	33 (67.3)
Antenatal steroids, any, No. (%)	63 (94.0)	17 (94.4)	46 (93.9)
Cesarean delivery, No. (%)	34 (50.7)	16 (88.9)	18 (36.7)*
Apgar score at 5 min, median (IQR)	8 (6, 9)	8 (7, 9)	8 (6, 9)
Days of FiO2 >0.21, median (IQR)	14 (2, 43)	24 (6, 52)	9 (1, 33)
Mechanical ventilation during NICU admission, No. (%)	29 (43.3)	12 (66.7)	17 (34.7)*
Duration of mechanical ventilation in days, median (IQR)	3 (2, 8.5)	4.5 (2.25, 7.75)	3 (2, 10)
Died before 36 weeks PMA, No. (%)	3 (4.4)	0 (0.0)	3 (6.1)
Moderate or severe BPD, No. (%)	18 (26.5)	9 (50.0)	9 (18.4)*
NEC stage II or greater, No. (%)	9 (13.4)	0 (0.0)	9 (18.4)
IVH grade II or greater, No. (%)	8 (11.9)	0 (0.0)	8 (16.3)
Sepsis, culture proven or clinically suspected, No. (%)	27 (40.3)	5 (27.8)	22 (44.9)

Supplemental table 1: Cohort characteristics of the 67 infants from the PulmonaRy Inflammation and glucocorticoiD sensitivity for the prediCTion of BronchoPulmonary Dysplasia (PRIDICT-BPD) study. Bronchopulmonary dysplasia was defined as the need for >21% oxygen for at least 28 cumulative days, and subsequently the severity of BPD was based on the need for oxygen or mode of respiratory support at 36 weeks postmenstrual age (PMA)[1]. Necrotizing enterocolitis (NEC) staging was defined according to Bell criteria[2,3] and IVH grading was defined according to Papile criteria[4]. Patient characteristics are shown for the entire cohort and for small for gestational age (SGA) and appropriate for gestational age (AGA) infants. Small for gestational age was defined as birth weight below the 2.3 percentile according to the Dutch reference curve[5]. * indicates statistically significant differences in outcomes between SGA and AGA infants (p-value < 0.05). IQR interquartile range.

**Patient characteristic**	**N=6**
Age, yrs, median (IQR)	48.5 (39.5 – 56.25)
Male sex, No. (%)	3 (50.0)

Supplemental table 2: Cohort characteristics of the 6 healthy controls.

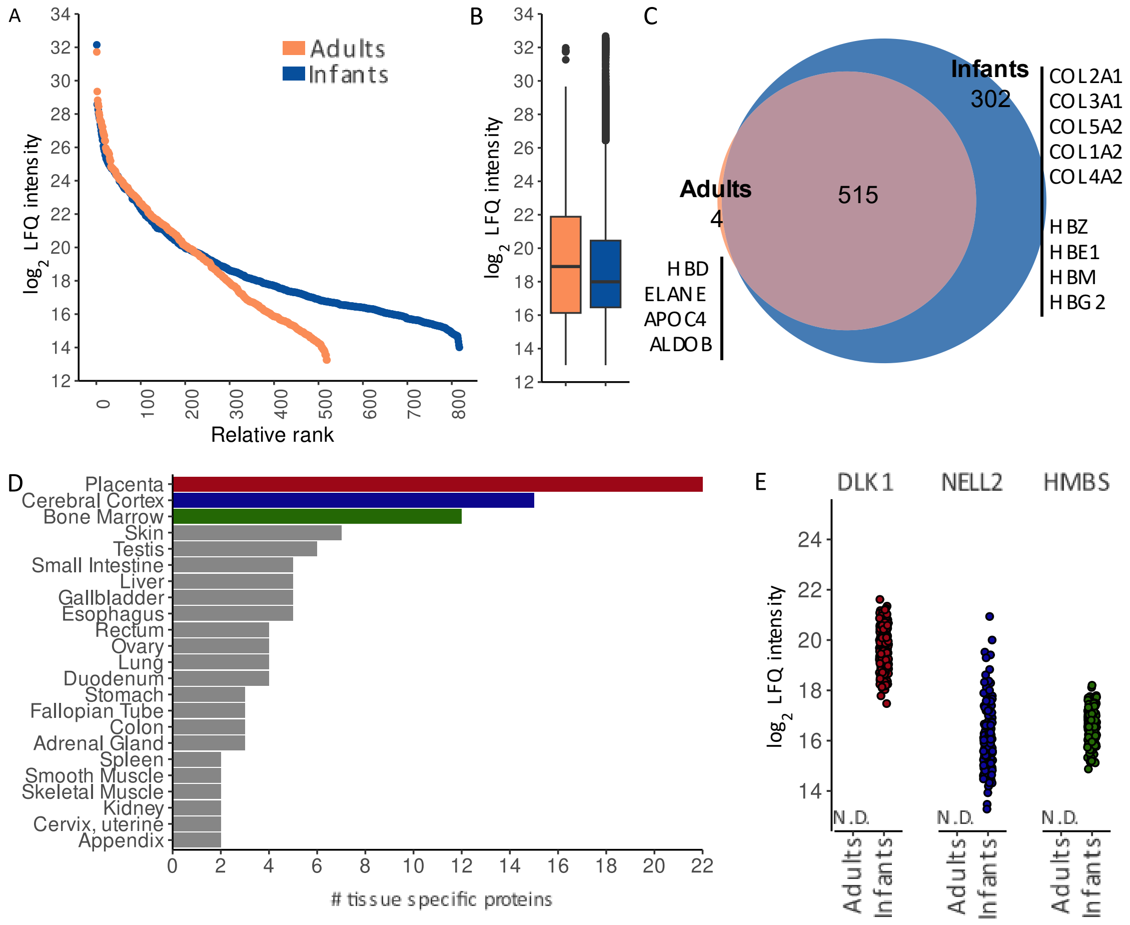

Supplemental figure 1: (A) Ranking of proteins quantified in adults (orange) and infants (blue) by label free quantification (LFQ)-intensity. (B) Boxplot representing the distribution of LFQ-intensity levels of all proteins shown in panel A. (C) Venn diagram representing the shared and unique proteins quantified in adults and infants. (D) Tissue-enrichment analysis performed on the 302 proteins unique to infants, with the top-3 tissues highlighted in red (placenta), blue (cerebral cortex) and green (bone marrow). (E) LFQ-intensity levels for protein delta homolog 1 (DLK1), porphobilinogen deaminase (HBMS) and protein kinase C-binding protein NELL2 (NELL2) in infants and adults.

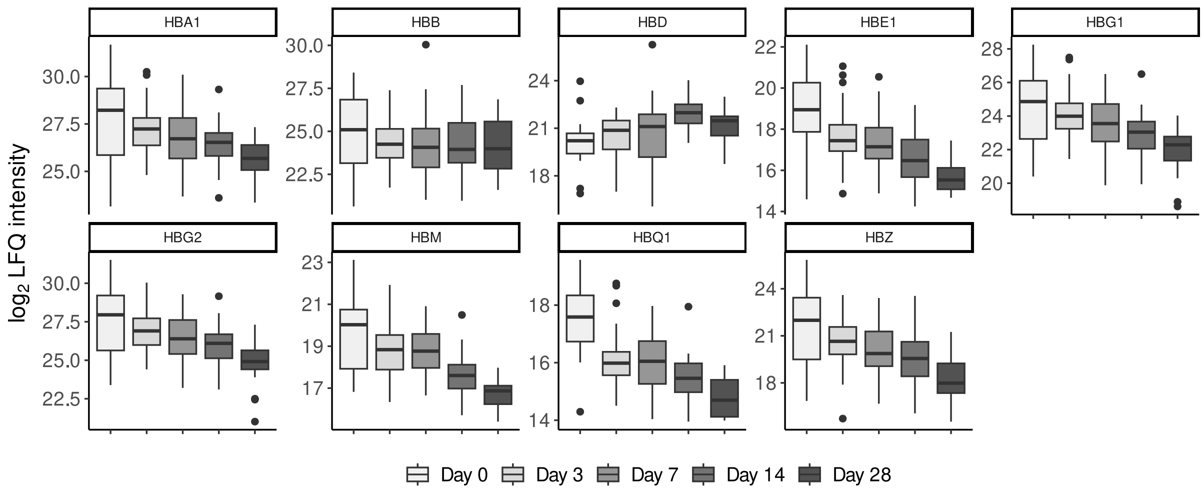

Supplemental figure 2: Protein levels of main hemoglobin subunits over postnatal age, based on prototypic peptides only. Hemoglobin subunit alpha (HBA1); Hemoglobin subunit beta (HBB); Hemoglobin subunit delta (HBD); Hemoglobin subunit epsilon (HBE1); Hemoglobin subunit gamma-1 (HBG1); Hemoglobin subunit gamma-2 (HBG2); Hemoglobin subunit mu (HBM); Hemoglobin subunit theta-1 (HBQ1); Hemoglobin subunit zeta (HBZ).

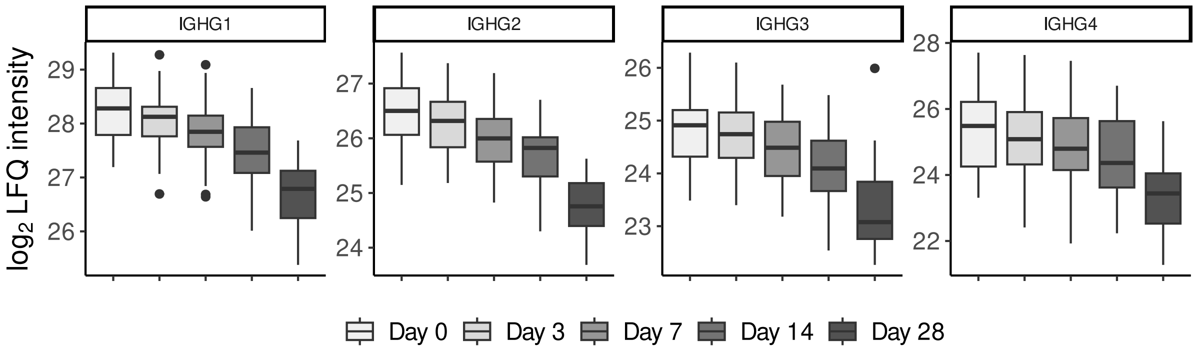

Supplemental figure 3: Protein levels of main immunoglobulin subunits over postnatal age, based on prototypic peptides only. Immunoglobulin heavy constant gamma 1 (IGHG1); Immunoglobulin heavy constant gamma 2 (IGHG2); Immunoglobulin heavy constant gamma 3 (IGHG3); Immunoglobulin heavy constant gamma 4 (IGHG4).

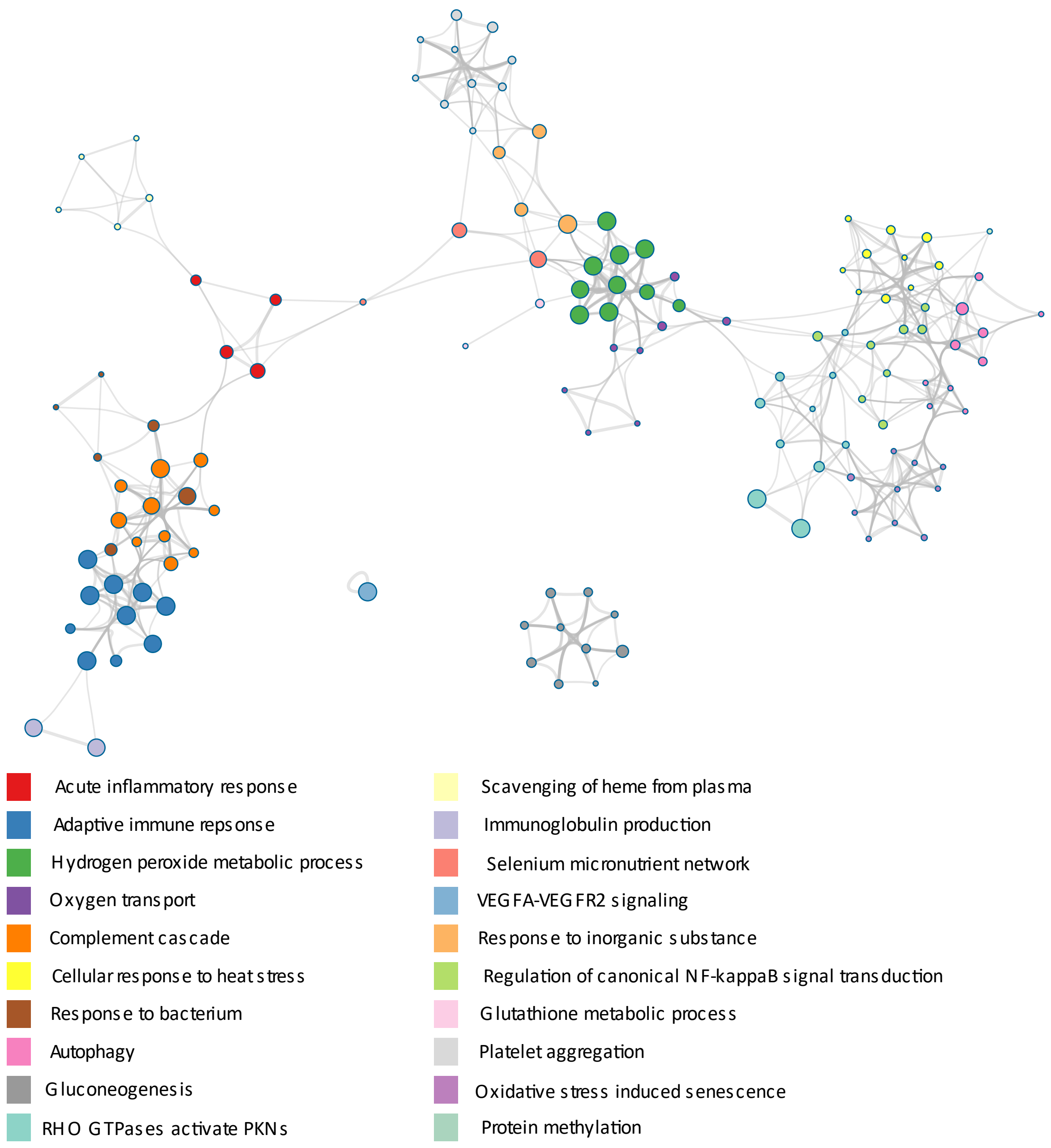

Supplemental figure 4: Metascape enrichment network visualization for results from proteins that show developmental increases and decreases with postnatal age colored by the enriched process in which all 314 proteins are involved. The color code in the networks represents the enriched biological processes. The network shows the interactions between the processes.

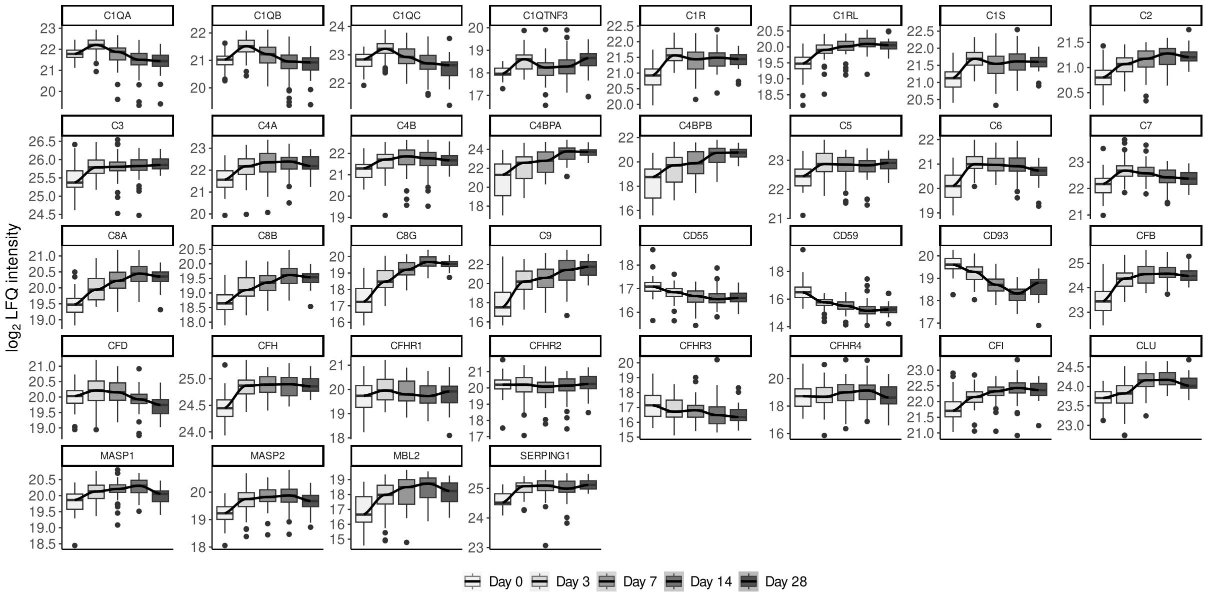

Supplemental figure 5: Protein levels of complement cascade proteins over postnatal age, based on prototypic peptides only. Black line represents the median protein level per timepoint used to determine the similarity score and effect size of the protein levels over time.

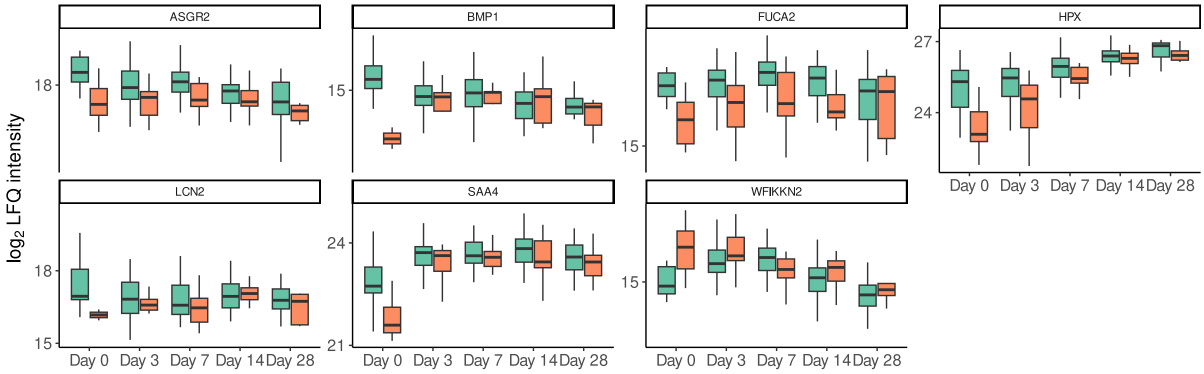

Supplemental figure 6: Protein levels over postnatal age for proteins with significant alterations in small for gestational age (SGA, orange) infants compared to appropriate for gestational age (AGA, green) infants in cord blood.

1. Jobe AH. The new bronchopulmonary dysplasia. Curr Opin Pediatr. 2011;23:167–72.

2. Neu J, Walker WA. Necrotizing Enterocolitis. N Engl J Med. 2011;364:255–64.

3. Bell MJ, Ternberg JL, Feigin RD, Keating JP, Marshall R, Barton L, et al. Neonatal necrotizing enterocolitis. Therapeutic decisions based upon clinical staging. Ann Surg. 1978;187:1–7.

4. Papile L-A, Burstein J, Burstein R, Koffler H. Incidence and evolution of subependymal and intraventricular hemorrhage: A study of infants with birth weights less than 1,500 gm. J Pediatr. 1978;92:529–34.

5. Hoftiezer L, Hof MHP, Dijs-Elsinga J, Hogeveen M, Hukkelhoven CWPM, van Lingen RA. From population reference to national standard: new and improved birthweight charts. Am J Obstet Gynecol. 2019;220:383.e1-383.e17.
